# Viscosupplementation with High Molecular Weight Hyaluronic Acid for Hip Osteoarthritis: An Updated Systematic Review and Meta-Analysis of Randomized Controlled Trials

**DOI:** 10.1055/s-0046-1819579

**Published:** 2026-04-22

**Authors:** Tales Pasqualotto, Eric Pasqualotto, Leonardo Salvatore Migliardi, Laura Lunelli, Letícia Oliveira Lunelli, Renan Vinicius Romano Martinelli

**Affiliations:** 1 Departamento de Medicina, Universidade Alto Vale do Rio do Peixe, Caçador, SC, Brazil; 2 Departamento de Cirurgia, Universidade Federal de Santa Catarina, Florianópolis, SC, Brazil; 3 Ortopedia e Traumatologia, Hospital Governador Celso Ramos, Florianópolis, SC, Brazil; 4 Departamento de Medicina, Universidade do Sul de Santa Catarina, Palhoça, SC, Brazil; 5 Ortopedia e Traumatologia, Hospital Municipal do Tatuapé, São Paulo, SP, Brazil; 6 Ortopedia e Traumatologia, Hospital Regional de São José Dr. Homero de Miranda Gomes, São José, SC, Brazil

**Keywords:** hip, hyaluronic acid, osteoarthritis, ácido hialurônico, osteoartrite, quadril

## Abstract

**Objective:**

To evaluate the efficacy and safety of high molecular weight hyaluronic acid (HMWHA) versus other therapies for hip osteoarthritis (OA) management.

**Methods:**

The present systematic review and meta-analysis followed the Preferred Reporting Items for Systematic Reviews and Meta-Analyses (PRISMA) guidelines. Randomized controlled trials (RCTs) comparing HMWHA versus other therapies (corticosteroids, platelet-rich plasma, saline, or low molecular weight hyaluronic acid) for hip OA treatment were included. Mean differences (MDs) or standardized mean differences (SMDs) were calculated for continuous outcomes with 95% confidence intervals (CIs).

**Results:**

Four RCTs were included, involving 823 patients with hip OA, of whom 408 (49.5%) were treated with HMWHA. The mean age of the patients was 60.1 (±10.21) years. No significant differences were observed between groups for pain (SMD −0.30 points; 95% CI −1.60 to 0.99), Lequesne index (MD 1.30 points; 95% CI −8.83 to 11.44), WOMAC total (MD −9.26 points; 95% CI −51.33 to 32.56), WOMAC stiffness (MD −0.93 points; 95% CI −12.30 to 10.45), WOMAC physical function (MD −0.15 points; 95% CI −7.24 to 7.60), and patient global self-assessment (MD −1.95 points; 95% CI −27.49 to 23.59).

**Conclusion:**

No significant differences were observed between HMWHA and other treatments regarding pain relief and functional recovery in patients with hip OA. However, further high-quality RCTs are needed to evaluate the HMWHA in the treatment of hip OA.

## Resumo

**Objetivo:**

Avaliar a eficácia e a segurança do ácido hialurônico de alto peso molecular (AHAPM) em comparação a outros métodos para o tratamento da osteoartrite (OA) do quadril

**Métodos:**

Esta revisão sistemática e metanálise seguiu as diretrizes Preferred Reporting Items for Systematic Reviews and Meta-Analyses (PRISMA). Foram incluídos estudos clínicos randomizados (ECRs) que compararam o AHAPM com outras terapias (corticosteroides, plasma rico em plaquetas, solução salina ou ácido hialurônico de baixo peso molecular) para o tratamento da OA do quadril. As diferenças médias (DMs) ou diferenças médias padronizadas (DMPs) de desfechos contínuos foram calculadas com intervalos de confiança (IC) de 95%.

**Resultados:**

Quatro ECRs foram incluídos, com um total de 823 pacientes com OA do quadril, dos quais 408 (49,5%) foram tratados com AHAPM. A idade média dos pacientes foi de 60,1 (±10,21) anos. Não foram observadas diferenças significativas entre os grupos em relação à dor (DMP, −0,30 pontos; índice de confiança [IC] 95%, −1,60–0,99), índice de Lequesne (DM, 1,30 pontos; IC 95%, −8,83–11,44), pontuação total do Western Ontario and McMaster Universities Osteoarthritis Index (WOMAC) (DM, −9,26 pontos; IC 95%, −51,33–32,56), rigidez segundo o WOMAC (DM, −0,93 pontos; IC 95%, −12,30–10,45), função física segundo o WOMAC (DM, −0,15 pontos; IC 95%, −7,24–7,60) e autoavaliação global pelo paciente (DM, −1,95 pontos; IC 95%, −27,49–23,59).

**Conclusão:**

Não foram observadas diferenças significativas entre o AHAPM e outros tratamentos em relação ao alívio da dor e à recuperação funcional em pacientes com OA do quadril. No entanto, mais estudos clínicos randomizados de alta qualidade são necessários para avaliação do AHAPM no tratamento da OA do quadril.

## Introduction

Hip osteoarthritis (OA) is a chronic, degenerative, and multifactorial joint disorder, affecting ∼ 6.4% of the global population and ranking among the leading causes of pain and disability. 1 2 It is marked by progressive cartilage deterioration and structural joint changes, driven by both mechanical and inflammatory mechanisms. 3 While various non-surgical treatments are available, many patients ultimately experience substantial functional decline, making hip arthroplasty the definitive therapeutic option. 1

Several non-surgical therapeutic approaches are recommended for managing hip OA, including non-pharmacological strategies such as exercise, weight control, and physiotherapy, 4 as well as analgesics and nonsteroidal antiinflammatory drugs (NSAIDs), which remain first-line treatments despite their associated risks. 1 4 Intra-articular injections of corticosteroids and hyaluronic acid (HA) are also employed, although their efficacy in pain reduction is variable. 4 5 Notably, HA viscosupplementation has garnered increasing attention, particularly with high molecular weight formulations such as hylan G-F 20, whose enhanced elasticity and viscosity closely mimic the properties of native synovial fluid. 1 6

High molecular weight hyaluronic acid (HMWHA) viscosupplementation has been investigated as a therapeutic option for hip OA, with studies reporting improvements in pain and function. 5 6 However, findings remain inconsistent regarding the optimal therapeutic approach for hip OA, largely due to methodological heterogeneity among studies. Therefore, the current systematic review and meta-analysis aims to update and synthesize evidence from randomized controlled trials (RCTs) on the efficacy and safety of HMWHA in the management of hip OA.

## Methods

The present systematic review followed the Preferred Reporting Items for Systematic Reviews and Meta-Analysis (PRISMA) guidelines. 7 The study protocol was registered in the International Prospective Register of Systematic Reviews (PROSPERO) 8 with registration number CRD420251123450.

### Search Strategy and Data Extraction

PubMed, Embase, and Cochrane Library were systematically searched from inception to January 2025, with the following search strategy: (“hylan G-F 20” OR “high molecular weight hyaluronic acid" OR HMWHA) AND (“hip osteoarthritis” OR “hip OA” OR HOA”. Aiming the inclusion of additional studies, references of the included articles and systematic reviews of the literature were evaluated. Two authors (T.P. and E.P.) independently extracted baseline characteristics and data outcomes following predefined search criteria. Three authors resolved disagreements by consensus (TP, EP, and RVRM).

### Eligibility Criteria

Studies with the following criteria were included: (1) RCTs; (2) comparing HMWHA and other therapies (corticosteroids, platelet-rich plasma [PRP], saline, or low molecular weight hyaluronic acid [LMWHA]); (3) comprising patients with hip OA; and (4) reporting at least 1 of the outcomes of interest. Studies with the following criteria were excluded: (1) non-RCTs; (2) populations of no interest; and (3) interventions of no interest.

### Endpoints and Definitions

Outcomes of interest were: (1) pain, (2) Lequesne index, (3) Western Ontario and McMaster Universities Osteoarthritis Index (WOMAC) total, (4) WOMAC stiffness, (5) WOMAC physical function, and (6) patient global self-assessment.

The outcome of overall pain was assessed using the main pain measure in each study (Visual Analog Scale [VAS] or WOMAC pain, with higher scores indicate worsening.

The WOMAC is a widely adopted questionnaire developed to evaluate pain, joint stiffness, and physical function in individuals with OA. Scores range from 0 to 96, with higher values reflecting greater disability.

### Risk of Bias Assessment

The Cochrane Collaboration tool for assessing risk of bias in randomized trials (Rob-2) was used to assess the quality of individual RCTs. 9 Each trial received a low, some concerns, or high risk-of-bias score across five domains: randomization process; deviations from the intended interventions; missing outcomes; measurement of the outcome; and selection of reported results. Two independent authors (TP and EP) performed the risk-of-bias assessment, with any disagreements resolved by consensus with the senior author. Publication bias was assessed through visual inspection of funnel plots. No quantitative analyses were made due to the small number of included studies (n < 10). 10

### Statistical Analysis

The treatment effects for continuous outcomes were compared using mean differences (MDs) or standardized mean differences (SMDs) with 95% confidence intervals (CIs). Heterogeneity was assessed with the Cochran Q-test and I ^2^ statistics; *p* -values < 0.10 and I ^2^ values > 25% were considered to indicate significance for heterogeneity. 11 Restricted Maximum Likelihood (REML) random-effects models were used for all endpoints. 12 To improve the robustness of the inference, 95% CIs were adjusted using the Hartung-Knapp method. Leave-one-out sensitivity analyses were performed to identify influential studies and their effect on the pooled estimates for pain outcomes. For data handling and conversion, the guidelines of the Cochrane Handbook for Systematic Reviews of Interventions were used. 13 Statistical analyses were performed using the R statistical software (R Foundation for Statistical Computing), version 4.4.1.

## Results

### Study Selection and Characteristics

**Fig. 1:**
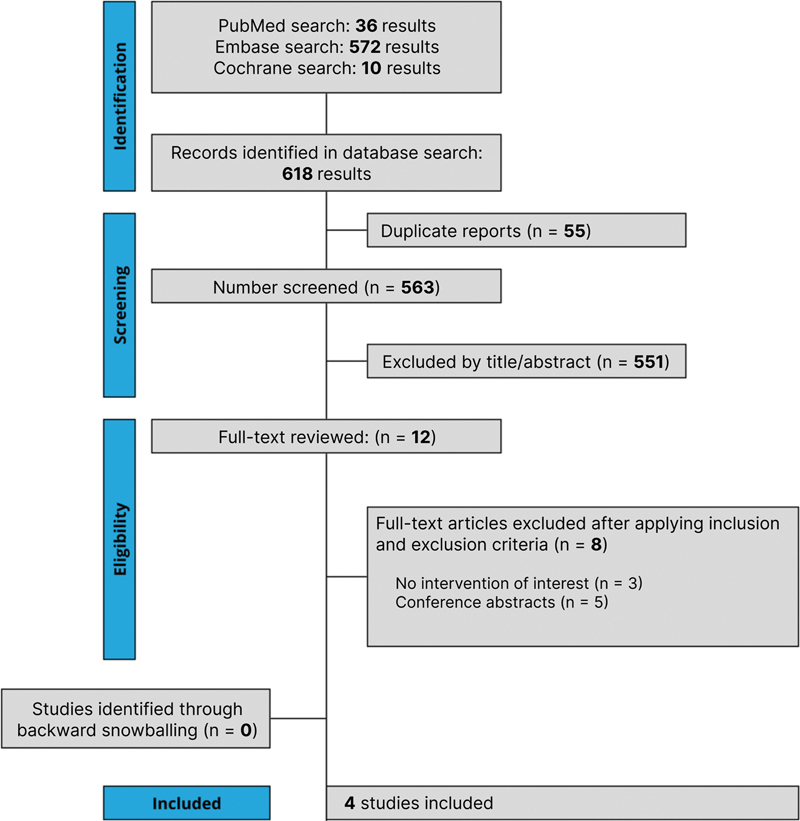
Preferred Reporting Items for Systematic Review and Meta-Analysis (PRISMA) flow diagram of study screening and selection.

The initial search identified 618 results, as illustrated in Fig. 1 . After removing duplicates and screening the studies by title and abstract, 12 full-text articles met the criteria for detailed evaluation. Among these, 4 RCTs were selected for inclusion, encompassing a total of 823 patients with hip OA, of whom 408 (49.5%) received HMWHA. 1 5 6 14 The duration of follow-up ranged from 6 months to 26 weeks. The mean age of the patients was 60.1 years, and 57.5% were female. Detailed characteristics of the studies and participants are presented in Table 1 .

**Table 1:** Characteristics of studies and participants included in the meta-analysis

Study	Country	Intervention	Control	Follow-up	Sample size IG/CG, n	Age IG/CG, years, mean ± SD	Gender M:F IG/CG, n	BMI IG/CG, kg/m ^2^ , mean ± SD	VAS score pretreatment (IG/CG), mean ± SD	Lequesne index pretreatment, mean ± SD
Spitzer 2010	USA	2 IA 2-mL injections of hylan G-F 20 (administered 2 weeks apart)	1 IA 2-mL injection of MPA (40 mg) followed by a sham injection 2 weeks later	26 weeks	156/156	59 ± 12/ 59 ± 11	75:81/76:80	29.3 ± 5.5 / 29.4 ± 6.0	NA	NA
Tikiz 2005	Turkey	Hylan G-F 20 (Synvisc 2.0 ml)	LMWHA solution (Ostenil 2.0 ml)	6 months	18/25	60.4 ± 9.6/ 58.8 ± 9.8	4:14/5:20	29.8 ± 3.9/28.7 ± 4.3	6.7 ± 1.7/7.2 ± 1.5	11.8 ± 3.3/ 11.4 ± 4.6
Brander 2019	Canada	Hylan G-F 20 single 6-mL injection	Phosphate-buffered saline (one 6-mL IA injection)	26 weeks	182/175	60.8 ± 10.0/ 59.8 ± 8.8	76:106/70:105	30.9 ± 14.2/29.1 ± 7.6	NA	NA
Nouri 2022	Ira	2.5 ml injection contained 50 mg linear fermentation source HMWHA	5 ml of autologous PRP	6 months	29/32	60.93 ± 4.54/ 58.22 ± 5.10	22:07/22:10	27.62 ± 2.25/27.72 ± 2.11	8.10 ± 1.18/ 7.63 ± 1.31	12.52 ± 2.34/ 12.20 ± 2.18

**Abbreviations** : BMI, body mass index; CG, control group; HMWHA, high molecular weight hyaluronic acid; IA, intra-articular; IG, intervention group; LMWHA, low molecular weight hyaluronic acid; MPA, methylprednisolone acetate; NA, not available; SD, standard deviation; USA, United States of America.

### Pooled analysis of all studies

**Fig. 2:**
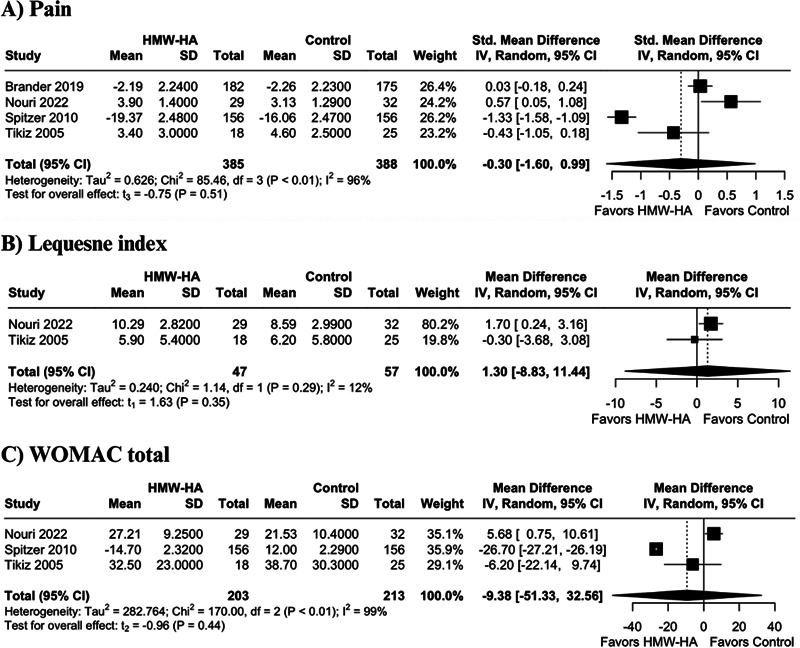
Forest plots of comparison between high molecular weight hyaluronic acid (HMWHA) versus other therapies. ( **A** ) Pain. ( **B** ) Lequesne index. ( **C** ) WOMAC total.

**Fig. 3:**
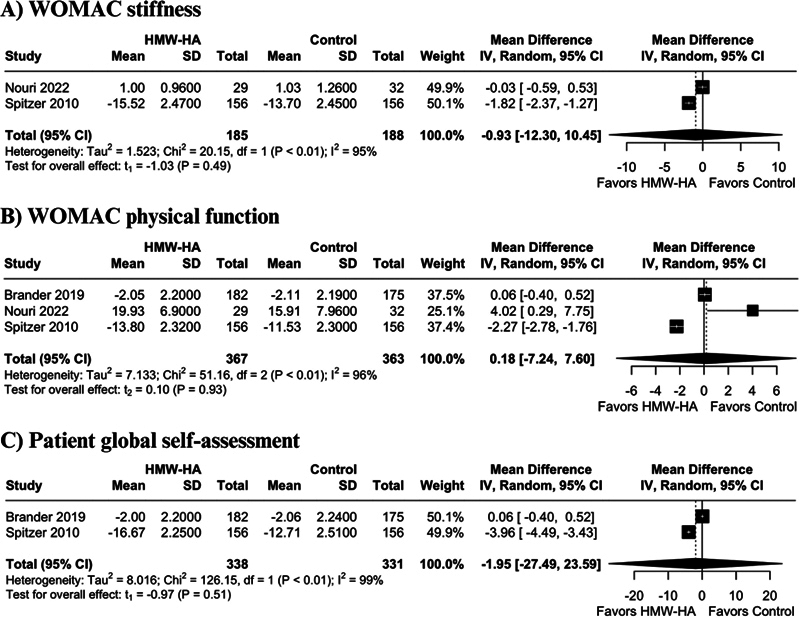
Forest plots of comparison between HMWHA versus other therapies. ( **A** ) Western Ontario and McMaster Universities Osteoarthritis Index (WOMAC) stiffness. ( **B** ) WOMAC physical function. ( **C** ) Patient global self-assessment.

There were no significant differences between HMWHA and other therapies for pain (SMD −0.30 points; 95% CI −-1.60 to 0.99; *p* = 0.51; I ^2^ = 96%; Fig. 2A ), Lequesne index (MD 1.30 points; 95% CI −8.83 to 11.44; *p* = 0.35; I ^2^ = 12%; Fig. 2B ), WOMAC total (MD −9.38 points; 95% CI −51.33 to 32.56; *p* = 0.44; I ^2^ = 99%; Fig. 2C ), WOMAC stiffness (MD −0.93 points; 95% CI −12.30 to 10.45; *p* = 0.49; I ^2^ = 95%; Fig. 3A ), WOMAC physical function (MD −0.18 points; 95% CI −7.24 to 7.60; *p* = 0.93; I ^2^ = 96%; Fig. 3B ), and patient global self-assessment (MD −1.95 points; 95% CI −27.49 to 23.59; *p* = 0.51; I ^2^ = 99%; Fig. 3C ).

### Sensitivity Analysis

In the leave-one-out sensitivity analysis, the results for the pain outcome remained stable. The leave-one-out sensitivity analysis plot is detailed in **Supplementary Fig. S1** .

### Risk of Bias

**Fig. 4:**
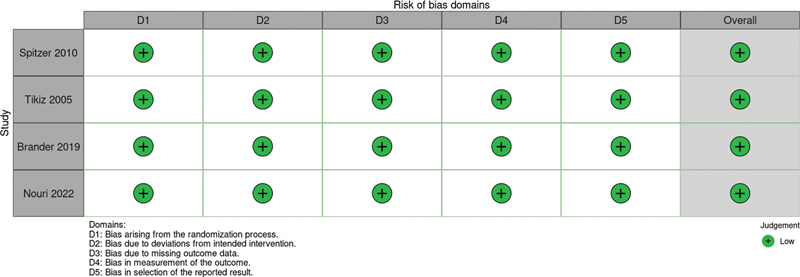
Critical appraisal of randomized controlled trials according to the Cochrane Collaboration's tool for assessing risk of bias in randomized trials.

Fig. 4 provides a detailed evaluation of each RCT included in the meta-analysis, revealing an overall low risk of bias across all studies.

## Discussion

In the present systematic review and meta-analysis of 4 RCTs including 823 patients with hip OA, we compared HMWHA to control interventions. We found no significant differences between groups in pain, Lequesne index, total WOMAC score, WOMAC stiffness, WOMAC physical function, or patient global self-assessment. Sensitivity analysis confirmed the robustness of the pain outcome.

Intra-articular injection of HMWHA aims to restore the properties of synovial fluid, reducing friction and providing symptomatic relief. 15 While extensively studied and widely used in knee OA, the application of HA in hip OA requires further investigation. Studies suggest that the molecular weight of HA is strongly correlated with its efficacy; accordingly, HMWHA exhibits superior properties, including prolonged intra-articular retention, robust chondroprotective effects, and enhanced modulation of inflammatory processes. 16 17 These effects are particularly significant given the relatively short intra-articular half-life of HMWHA, ∼ 8.8 days, which contrasts with the sustained clinical benefits observed. This discrepancy is explained by HMWHA's biological mechanisms, such as inducing endogenous HA synthesis by synovial fibroblasts and modulating inflammatory cytokines over the medium to long term. 17 Furthermore, formulations like Sinovial HL (IBSA Farmaceutici Italia Srl), which combine high and low molecular weight fractions, have demonstrated efficacy in treating mild to moderate hip OA, reinforcing the therapeutic potential of HMWHA in this disease. 15

The efficacy of HMWHA in the treatment of hip OA remains controversial. Richette et al. found no clinically significant benefit from a single intra-articular injection of HA (Adant; average molecular weight 900,000 Da) compared with placebo, suggesting that a single administration may be insufficient to achieve sustained therapeutic effects. 18 In contrast, Spitzer et al. reported that hylan G-F 20 produced clinically meaningful improvements in pain and function scores, particularly in patients with more advanced disease, with effects maintained for up to six months compared with methylprednisolone acetate. 1 Similarly, Tıkız et al. 6 observed that both hylan G-F 20 and LMWHA led to significant pain reduction and functional improvement through the sixth month, with no significant differences between the two groups, indicating comparable efficacy across molecular weights when administered in a three-dose weekly regimen. Conversely, Brander et al. 5 found no significant differences between a single 6 mL hylan G-F 20 injection and placebo in any of the clinical outcomes assessed.

Recently, PRP has emerged as a promising biologic therapy for hip OA, targeting inflammation modulation and joint tissue repair. Nouri et al. compared HA, PRP, and a combination of PRP with HA in patients with mild to moderate hip OA. They found that while all groups experienced significant improvements in WOMAC, VAS, and Lequesne scores up to six months, the PRP and PRP + HA groups demonstrated superior outcomes compared with the HA-only group, particularly in activities of daily living and total Lequesne scores, with statistically significant differences observed between the second and sixth months. 14

A meta-analysis by Gazendam et al. 19 demonstrated that intra-articular HA injections, whether combined with PRP or not, offered no clinically meaningful advantage over placebo in hip OA, and no significant differences were observed between LMWHA and HMWHA. In contrast to our findings and to the previous meta-analysis by Gazendam et al. 19 in hip OA, HA injections have shown benefits in knee OA, with some evidence suggesting that HMWHA may outperform LMWHA in this setting. The mechanisms underlying this apparent superiority in knee OA remain unclear, but it has been proposed that the higher viscosity of HMWHA enhances its lubricating capacity. The hip's spherical anatomy, as opposed to the relatively flatter joint surfaces of the knee, might diminish the relevance of this property in hip OA; however, this notion currently lacks empirical support.

This study has several limitations. First, the small number of included studies limits the generalizability of our findings. Second, there was substantial statistical heterogeneity across analyses, which may affect the robustness of the results. Therefore, we adjusted the 95% CIs using the Hartung–Knapp method to improve the robustness of the random-effects inference. Third, the use of different control groups among the included trials introduces variability that complicates direct comparisons. Finally, the inability to perform subgroup analyses according to OA severity or to assess the influence of confounding variables on outcomes restricts the depth of our conclusions.

## Conclusion

In this systematic review and meta-analysis, no significant differences were observed between HMWHA and other treatments regarding pain relief and functional recovery in patients with hip OA. However, further high-quality RCTs are needed to evaluate the HMWHA in the treatment of hip OA.
